# Cardiac Arrest and Gastrointestinal Bleeding: A Case of Medical Heuristics

**DOI:** 10.1155/2016/9621390

**Published:** 2016-06-06

**Authors:** Tokunbo Ajayi, Jerome Okudo

**Affiliations:** ^1^Department of Internal Medicine, Johns Hopkins University, 55 Cedar Lane, Columbia, MD 21044, USA; ^2^School of Public Health, University of Texas, 1200 Pressler Street, Houston, TX 77030, USA

## Abstract

Insufficient clinical data from patients is a major cause of errors in medical diagnostics. In an attempt to make a diagnosis, initial clinical information provided to the physician may be overly relied on as the only information required in making diagnosis leading to anchoring. Failure to rely on differential diagnoses in spite of new signs and symptoms or rethinking of initial hypothesis may lead to fixation on a certain diagnosis, which may lead to significant morbidity and mortality. In the event that there is an anchoring heuristic, like in our patient, it is important to consider differential diagnoses; however, it is not wrong to rely on some form of anchor. We report a case of a 62-year-old male with a history of multiple medical conditions and a history of acetaminophen overdose who presented to the hospital with large amounts of coffee ground emesis. He was subsequently transferred to the liver transplant center on discovery that he was in fulminant hepatic failure and died two days later in spite of aggressive medical treatment.

## 1. Introduction

Heuristics are rules or methods that enable problems to be solved quickly [1]. They are mental shortcuts that make cognitive reasoning or decision making easy [1]. Anchor and pattern recognition are important in a busy health facility because of the need for quick diagnosis, resources management, and the need to make quick decisions to save a patient's life [2]. Pattern recognition is very helpful when faced with complex decisions; it is also useful in starting the diagnostic process in spite of incomplete data [3].

However, overrelying on these shortcuts can be dangerous and lead to medical error [3]. We discuss an interesting case of cardiac arrest and gastrointestinal bleeding to buttress this point.

Out of hospital cardiac arrest (OHCA) is a common but unfortunate occurrence that physicians and family members encounter on a daily basis. The etiology is cardiac or noncardiac [4]. It might also be stratified into witnessed or unwitnessed as well as shockable or unshockable rhythm. This classification has informed our response to OHCA with the increased use of defibrillators and emergency responders and bystanders' use of CPR. CPR has led to increase in OHCA survival from 5.7% in 2005-2006 to 10.3% in 2014. The Utstein survival report in 2014 from the Cardiac Arrest Registry to Enhance Survival (CARES) showed OHCA is usually due to cardiac reasons (39445 arrests) as compared to noncardiac (7592 arrests) [4]. However, the etiology of OHCA can be difficult to establish at initial presentation where there is paucity of information surrounding the cardiac arrest and where initial laboratory data may be misleading [5]. This may lead to physician anchoring during the initial assessment and lead to delay in adequate management of patients [5, 6]. We present a case of OHCA who was thought to be due to hypervolemia from gastrointestinal bleeding and later found to have significant acetaminophen (APAP) overdose leading to hyperkalemia and OHCA. This case illustrates the danger of APAP overdose as a silent killer as well as the dilemma faced by a physician when he has initial limited clinical information during initial evaluation of patients.

## 2. Case Presentation

A 62-year-old man with a history of COPD, chronic back pain due to spinal stenosis on narcotics, NSAIDS and acetaminophen, depression, past cocaine use, and acetaminophen overdose/misuse presented with large amounts of coffee ground emesis and subsequent pulselessness. His wife, a nurse, began CPR with PEA arrest lasting 10 minutes with return of spontaneous circulation (ROSC) on arrival to the emergency department (ER). The patient was intubated on arrival to the ER; his vital signs included blood pressure of 79/37 mmHg, pulse of 52 beats per minute, temperature of 96.2 F, and pulse oximetry of 99% on 60% oxygen.

He was pale, unresponsive with 3 mm responsive pupil, intubated with good breath sounds bilateral and bradycardic with normal S1 and S2, no murmur rubs or gallops. His abdominal examination was unremarkable. His laboratory data was significant for white blood cell count of 18 and hemoglobin and hematocrit (H & H) of 8.7 and 27. His baseline platelets were 262. Prothrombin time was 39.3, with INR of 4.4, sodium 143, potassium 8.1, bicarbonate 11, chloride 108, BUN 26, creatinine 3, total bilirubin 1.7, direct bilirubin 0.7, and alkaline phosphatase 188. Creatine kinase was 3135. Initial lactic acid was 16 and a repeat was 16.6. Troponin was 0.08 and repeat was 0.11. Initial arrest etiology was thought to be secondary to hypovolemic shock from blood loss because of his initial presentation of hematemesis and laboratory result, but the degree of anemia did not explain his cardiac arrest. However, toxicology report was positive for benzodiazepines and cocaine and his acetaminophen level was greater than 300. Arterial blood gas was pH 6.7/partial pressure of carbon dioxide (pCO2) 69/partial pressure of oxygen (pO2 98) and he was started on a bicarbonate gamma glutamyl transferase with pH improving to 7.3.

Other etiologies considered at this point included drug overdose and coronary vasospasm from cocaine use; however no EKG changes were observed. He was transfused with packed red blood cell (PRBC). However, the patient went into cardiac arrest again and had return of spontaneous circulation (ROSC) after 10 minutes of CPR, boluses of normal saline, epinephrine, atropine, amiodarone, and dopamine were given. He also received 2 doses of 30 mg kayexalate, calcium chloride, an ampoule of D50W, and 10 units of insulin. At this juncture in the patient hospital course, after the poor response with transfusion, he was thought to possibly have acute liver failure in the setting of acetaminophen overuse after further history from his wife revealed that he had increased the dosage and frequency of acetaminophen use in the last few days due to worsening chronic back pain. He was given IV acetylcysteine (NAC) 20 g stat and 10 g over continuous infusion over 16 hours. He was also given pantoprazole drip. He received 5 units of PRBC and 3 units of fresh frozen plasma (FFP). Bedside echocardiography showed no significant wall motion abnormality or pericardial effusions. Therapeutic cooling protocol was started. A CT brain showed global cerebral edema and a CT abdomen was consistent with small bowel ischemia, small bowel pneumatosis, extensive mesenteric venous gas, and extensive portal venous gas.

There was prominence of small bowel loops without frank bowel obstruction. There was underlying diffuse low accentuation to liver, which can be seen in fatty infiltration and/diffuse edema. He was transferred to a transplant hepatology center for liver transplant; however due to his overall clinical condition with multisystem organ failure and possible ongoing substance use (cocaine and narcotics), transplantation was declined and he was therefore managed medically. There was an extensive discussion with the family by the palliative care team concerning the goal of care based on his prognosis and the family consensus was that there was no need for further aggressive therapy. Patient died 2 days after presentation due to cardiopulmonary arrest.

## 3. Discussion

This case posed a few challenges to the admitting physician. Firstly, when patients present in a comatose state, clinical history surrounding their presentation is usually scarce and if available is from an unverified source. Sometimes, this information is nonexistent. This leaves the healthcare giver little to no information to work with to make a lifesaving decision. This case illustrates that, though some information could be garnered from the patient's wife, it was incomplete, as she did not provide upfront the history of significant APAP use by the patient in the last few days prior to his presentation. This significant increase in use of APAP was due to her husband's worsening chronic back pain. However, she was able to give the history that the patient was experiencing GI bleed in the last few days prior to presentation in the hospital. This seems helpful but became anchoring data to the physician who had little information to go on. Anchoring can be useful in medicine but if relied upon for so long could lead to error [7]. Anchoring is defined as bias that describes the common human tendency to rely too heavily on the first piece of information offered (the “anchor”) when making decisions [7–9]. During decision-making, anchoring occurs when individuals use an initial piece of information to make subsequent judgments [8]. In this case, the initial history of GI bleed with the first laboratory value of significant low hemoglobin and hematocrit led to the belief that the patient's cardiac arrest was due to hypovolemia from significant blood loss. These led to the urgent GI consult for endoscopy management. The patient was resuscitated as he went into cardiac arrest.

As laboratory data became available and he was noted to have significant hyperkalemia (potassium of 7), the initial diagnosis was questioned with more analysis of the data. The process of further questioning of the relatives (patient's wife) and the availability of laboratory results (APAP level >300 and elevated transaminases) led to important data that helped reshape the diagnosis and subsequent investigations and management.

The importance of precise decision making cannot be overemphasized; however there should be consideration of different settings: emergency, outpatient, inpatient, or surgery as these affect decision making and leave room for heuristics [9, 10]. The same type of reasoning does not apply to formal or inductive reasoning which allows for varying levels of certainty when making diagnosis [1, 10, 11]. A study has shown that Bayesian reasoning does not have significant impact in the estimation of probability. It showed that in spite of the use of Bayesian reasoning, physicians would still utilize heuristics in making diagnoses [1, 12]. This explains therefore that heuristics are important tools in medicine [12]. Another study showed that, whether medical students or physicians, heuristics were still utilized in spite of medical knowledge available to these cohorts [12]. This also shows that heuristic processes are not consciously made and are circumstantial and physicians do not recognize them when they are being made [13].

The case discussed is significant because of the importance of decision making in medical practice. It illustrates the biases that occur in this process. In our patient, we were biased in two ways: anchoring and pattern recognition [1, 11, 12]. Research has explained that neurocognitive decision-making occurs in two ways: a fast and otherwise reflexive system known as system 1 and a slow, deliberate system, system 2 [1, 13]. System 1 is characteristic of the following: garnered knowledge with little brain effort while system 2 requires a lot of effort. In an emergency setting like in our patient, system 1 was more applicable because it is easier hence our initial diagnosis [1–3, 13]. In the management of our patient, it was necessary to learn when to switch from a quick approach to a slower approach. However, considering the slowness of system 2 and the fact that it is missing from the medical curriculum, system 1 was more applicable in the emergency setting especially because of pressure and the need for short cuts in daily medical practice which heuristics offers in decision making [13–15].

Anchoring led us to hold on to the information given to us and work with it until laboratory investigations and an additional history of APAP use were made available. The lesson to be gleaned from this is to challenge the diagnoses in circumstances like this as it could lead to premature closure where there was a focus on a diagnosis without considering differential diagnoses. In this case, the physician cannot be faulted because of the life threatening nature and the rapidity of the presentation. Considering pattern recognition, when certain points in a case present in a pattern, it causes an almost reflex response for a case to be perceived and this leads to anchoring bias [2–5]. Initially, there is a bottom up approach where the physician's decision is driven by visual cues; however, with more clinical information at the physician's disposal, the process becomes clearer [1, 15]. However, in a top down approach, there is a focus on achieving a goal and this is dependent on the physician's expected outcomes [1–3]. It is imperative to have an interplay of both approaches. This interplay depends on the physician's clinical acumen and experience. The accuracy of diagnosis is not dependent on the content of the case or mastery of the case but may be dependent on approach. This interplay dependent on the content of the case or mastery of the case but may be dependent on approach. Tools that measure these heuristics are yet to be developed; however, the importance of involving patients in their management cannot be overemphasized especially if they are involved from a cognitive point of view in the diagnostic process. There should be an emphasis on improving heuristic strategies.

## 4. Conclusion

Careful evaluation of patients is very important to make an accurate diagnosis. Heuristics are useful tools but they are more effective when a balance between systems 1 and 2 exists, that is, heuristics versus Bayesian methods. It is also important to know where to draw the line in waiting to make the switch between systems 1 and 2 in anchor heuristics. The emphasis on the application of anchor heuristics should be key in patients because a study has shown that a simple framework of heuristics might not be representative of the trueness and advantages of heuristics such as anchoring. There must be interplay between evidence based medicine and problem based learning. A balance between the two is required in making diagnosis. It is imperative to create an environment or a culture that reduces stress for the physician, to always use differential diagnoses even when the diagnosis seems obvious, and to train humble physicians who are willing to listen to patients and other team members so that they can revisit the evidence when they are questioned about their diagnosis or management of patients. In addition, it is important to make clinical decisions and diagnoses based on evidence rather than experience.

## References

[1] Jacob K. S. (2015). The challenge of medical diagnosis: a primer on principles, probability, process and pitfalls. *The National Medical Journal of India*.

[2] Dredla B., Freeman W. D. (2016). Ehrlichia meningitis mimicking aneurysmal subarachnoid hemorrhage: a case study for medical decision-making heuristics. *The Neurohospitalist*.

[3] Phang S. H., Ravani P., Schaefer J., Wright B., McLaughlin K. (2015). Internal Medicine residents use heuristics to estimate disease probability. *Canadian Medical Education Journal*.

[4] https://mycares.net/sitepages/presspublications.jsp.

[5] Jasper F., Ortner T. M. (2014). The tendency to fall for distracting information while making judgments: development and validation of the objective heuristic thinking test. *European Journal of Psychological Assessment*.

[6] McLaughlin K., Eva K. W., Norman G. R. (2014). Reexamining our bias against heuristics. *Advances in Health Sciences Education*.

[7] van den Berge K., Mamede S. (2013). Cognitive diagnostic error in internal medicine. *European Journal of Internal Medicine*.

[8] Nam C. H., Jeon B. (2015). Lesson from a case of cervical meningioma misdiagnosed as parkinsonism. *Neurologist*.

[9] Elia F., Aprà F., Verhovez A., Crupi V. (2016). ‘First, know thyself’: cognition and error in medicine. *Acta Diabetologica*.

[10] Lucchiari C., Pravettoni G. (2013). The role of patient involvement in the diagnostic process in internal medicine: a cognitive approach. *European Journal of Internal Medicine*.

[11] Berner E. S., Graber M. L. (2008). Overconfidence as a cause of diagnostic error in medicine. *American Journal of Medicine*.

[12] Rylander M., Guerrasio J. (2015). Heuristic errors in clinical reasoning. *The Clinical Teacher*.

[13] Gorini A., Pravettoni G. (2011). An overview on cognitive aspects implicated in medical decisions. *European Journal of Internal Medicine*.

[14] Manesh R. S., Dixson J., Kohlwes R. J. (2015). Don't drop the anchor. *Journal of General Internal Medicine*.

[15] Dhaliwal G. (2011). Going with your gut. *Journal of General Internal Medicine*.

